# *In Silico* Investigation of Traditional Chinese Medicine Compounds to Inhibit Human Histone Deacetylase 2 for Patients with Alzheimer's Disease

**DOI:** 10.1155/2014/769867

**Published:** 2014-06-23

**Authors:** Tzu-Chieh Hung, Wen-Yuan Lee, Kuen-Bao Chen, Yueh-Chiu Chan, Cheng-Chun Lee, Calvin Yu-Chian Chen

**Affiliations:** ^1^Department of Biomedical Informatics, Asia University, Taichung 41354, Taiwan; ^2^School of Medicine, College of Medicine, China Medical University, Taichung 40402, Taiwan; ^3^Department of Neurosurgery, China Medical University Hospital, No. 2, Yude Road, North District, Taichung 40447, Taiwan; ^4^Department of Anesthesiology, China Medical University Hospital, Taichung 40447, Taiwan; ^5^Research Center for Chinese Medicine & Acupuncture, China Medical University, Taichung 40402, Taiwan; ^6^Human Genetic Center, Department of Medical Research, China Medical University Hospital, Taichung 40447, Taiwan

## Abstract

Human histone deacetylase 2 (HDAC2) has been identified as being associated with Alzheimer's disease (AD), a neuropathic degenerative disease. In this study, we screen the world's largest Traditional Chinese Medicine (TCM) database for natural compounds that may be useful as lead compounds in the search for inhibitors of HDAC2 function. The technique of molecular docking was employed to select the ten top TCM candidates. We used three prediction models, multiple linear regression (MLR), support vector machine (SVM), and the Bayes network toolbox (BNT), to predict the bioactivity of the TCM candidates. Molecular dynamics simulation provides the protein-ligand interactions of compounds. The bioactivity predictions of pIC50 values suggest that the TCM candidatesm, (−)-Bontl ferulate, monomethylcurcumin, and ningposides C, have a greater effect on HDAC2 inhibition. The structure variation caused by the hydrogen bonds and hydrophobic interactions between protein-ligand interactions indicates that these compounds have an inhibitory effect on the protein.

## 1. Introduction

Alzheimer's disease (AD) is a neuropathic degenerative disease in which patients will gradually suffer a loss of memory, language, intellect, motor action, and even life. In 2010, it was reported that about 36 million people worldwide suffered from AD [1]. The medical cost of this condition was predicted to be approximately 604 billion USD in 2010 [2]. This huge medical expense becomes a great social burden to an aging society.

Recently, it has been found that Tau protein [3], amyloid-*β* peptides [4], and human histone deacetylase (HDAC) are major factors in the causation of AD [5]. Human histone deacetylase 2 (HDAC2) is the protein expressed by* HDAC2* gene. Some reports have pointed out that* HDAC2* is over expressed in AD patients and that this gene negatively regulates memory [6–10]. There are also some references indicating that blocking the* HDAC2* gene could be a treatment for AD; furthermore, it has been shown to decrease amyloid-*β* peptides in mice [5, 11, 12]. HDACs catalyze the acetyl moiety, removing it from the lysine residues of proteins and regulating the level of protein acetylation [13]. The inhibition of* HDAC2* has been identified as a mechanism for treating cancer and developing histone deacetylase inhibitors (HDACi) [14]. As shown above, this inhibition mechanism could also be a model for the treatment of AD [6].

Some HDACi studies have indicated a role for chromatin remodeling increasing histone acetylation and enhancing synaptic plasticity and learning behaviors [15–17]. The clinical application of nonselective HDACi in cancer has shown a range of side effects [18, 19]. Suberoylanilide hydroxamic acid (SAHA or vorinostat) is a potent HDACi. SAHA binds to the active site of HDAC where it acts as a chelator for Zinc [13]. SAHA could cross the blood-brain barrier and decrease amyloid *β* peptides and treat AD and Huntington's disease (HD) by changes in histone acetylation in the brain [20–22].

Computer-aided drug design (CADD) is an* in silico* simulation technique for screening novel drug-candidate compounds by structure and prediction of biological activity. The two major application areas of CADD are structure-based drug design and ligand-based drug design. In comparison with traditional drug design, CADD has the advantages of both greater speed and lower cost. We used CADD for molecular simulation based on structure-based drug design, ligand-based drug design, and molecular dynamics [23–28].

Recently, an understanding of personalized medicine and biomedicine has been attracting more and more attention [29]; this department of knowledge could analyze regional diseases [30], clinical diagnosis cases, and disease associated mutations [31]. Traditional Chinese Medicine (TCM) plays an important role in Asia, especially in China, Taiwan, Korea, and Japan. The TCM Database@Taiwan (http://tcm.cmu.edu.tw/) [32] is the largest Traditional Chinese Medicine database in the world. This database contains 2D chemical structures, 3D chemical structures, bioactivity, and molecular information of 61,000 compounds used in Traditional Chinese Medicine. Since 2011, there have been successful discoveries in novel lead compounds from the TCM Database@Taiwan [33–35], including compounds for the putative treatment of AD [36], Parkinson's Disease [37], insomnia [38], pigmentary disorders [39], and even antivirals [40–44]. Due to the application system of the website [45] and cloud computing platforms [46], the TCM Database@Taiwan is exceptionally helpful for TCM applications and drug design.

In this study, we screen a possible lead compound against HDAC2 from the TCM Database@Taiwan. We use the computational techniques of docking, screening, and ligand-based methods to predict the bioactivity of the selected ligands. Finally, we apply molecular dynamics (MD) simulation to investigate variation from the protein-ligand interactions that may contribute to the evaluation of the effect of HDAC2 inhibition.

## 2. Materials and Methods

### 2.1. Data Set

Because the disorder protein plays an important role in drug design, the protein sequence should be submitted to the Database of Protein Disorder (DisProt, http://www.disprot.org/) for disorder prediction [47]. The result of prediction could help define the character of docking site and the efficacy of drug interaction.

A total of 61,000 TCM compounds were downloaded from the TCM database (http://tcm.cmu.edu.tw/). The HDAC2 (PDB ID: 3MAX) crystal structure was obtained from RCSB protein data bank. Based on the research, the SAHA was used as a control [48]. Accelrys Discovery Studio 2.5 (DS 2.5) was used to perform the molecular simulations.

### 2.2. Molecular Docking

LigandFit is a receptor-rigid docking algorithm program in Discovery Studio 2.5 (DS 2.5). The docking simulation was performed using LigandFit [49] module to dock SAHA and TCM compounds to HDAC2 in the force field of CHARMm [50]. The compounds downloaded from the TCM Database were docked into the SAHA binding site of HDAC2, which was identified from previous research [48].

### 2.3. Ligand-Based Prediction

We used the SVM, MLR, and BNT approaches as activity prediction models to predict the bioactivity of the TCM compounds. The SVM model, which utilized a nonlinear mapping technique, was built using LibSVM [51]. The MLR, using linear evaluation, was established by MATLAB. The BNT is a directed graphical model based on the study of Bayes probability and network cluster in MATLAB. Fifty compounds [52] were divided randomly into the predictive models as a training set of ten compounds and test set for the other compounds. These compounds were drawn by ChemBioOffice and modified using the prepare ligand module in DS 2.5. The bioactivity (IC50) data of the compounds were converted to pIC50 by the QSAR program and their molecular descriptors were evaluated by calculate molecular properties from 552 different descriptors in Discovery Studio 2.5. The genetic function approximation (GFA) algorithm is based on a genetic algorithm to search all possible QSAR models and use a square correlation coefficient (*R* ^2^) to estimate the best representative molecular descriptors [53]. After the above processes, the bioactivity of TCM de novo compounds can be predicted through MLR, SVM, and BNT model by MATLAB and LibSVM.

### 2.4. Molecular Dynamics Simulation

The ligands must be reprepared by using SwissParam (http://swissparam.ch/) [54] before applying MD simulation based on the reference force field [55] of GROMACS 4.5.5 [56]. The HDAC protein combines with ligands as the complex in the buffer (or solution) simulation box. This cubic box provided a minimum distance of 1.2 Å from the complex and was solvated with the TIP3P water model in which sodium and chloride ions were added to neutralize complex charges. The complex was minimized with the steepest descent method for 5,000 steps. The last structure of minimization was transferred for MD simulation. The calculations for electrostatic interactions were based on the particle-mesh Ewald (PME) method [57]. In the PME method, each time step was 2 fs and there were 20,000,000 iterations. Equilibration under the 10 ps constant temperature (NVT ensemble) was based on the Berendsen weak thermal coupling method. The total simulation time for the MD was 40 ns. MD trajectories, RMSD, energy variations of the complex, and the secondary structure database (DSSP) were analyzed using a series of protocols in GROMACS.

## 3. Result and Discussion

### 3.1. Molecular Docking

The results of molecular docking are ranked according to the docking score. We selected the pdb data of these docking poses and removed the data that lacked any of the important amino-acids of HDAC2, such as Tyr29, Met35, Phe114, Leu144, Gly154, Phe155, Asp181, His183, Phe210, Asp269, and Leu276, which were identified by [48]. His142 was identified as the active site in HDAC2 based on Uniprot (http://www.uniprot.org/) data. The top twenty ligands were ranked according to the docking score and used for ligand-based predictions of their bioactivity.

### 3.2. Ligand-Based Prediction

**Figure 1:**
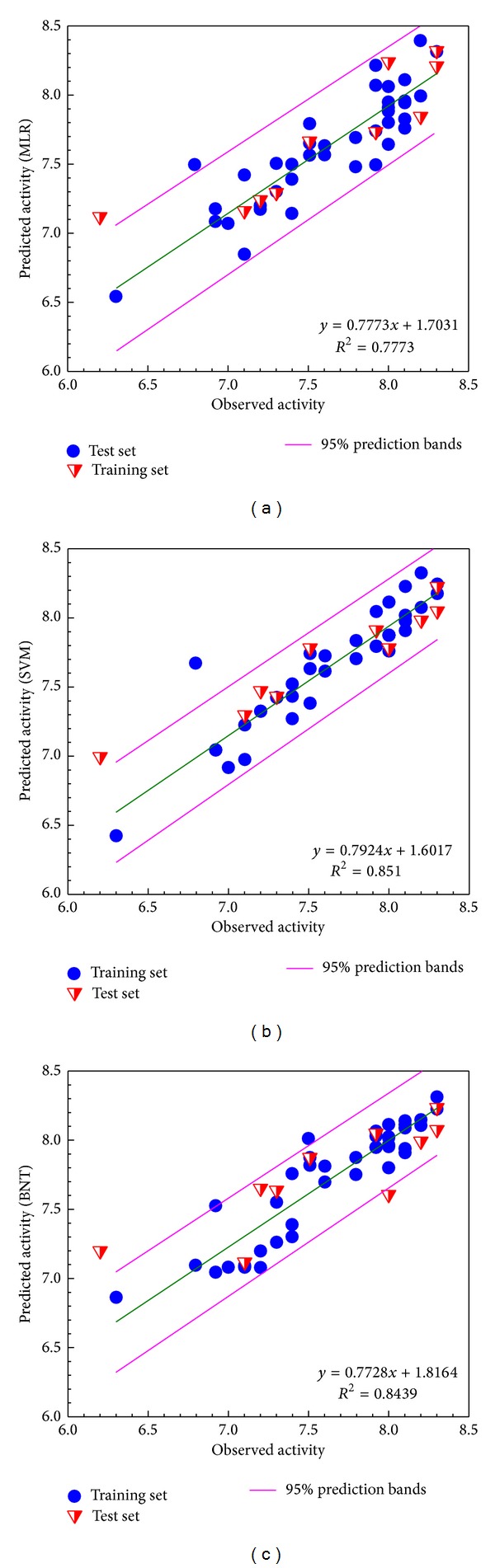
Relation of observed activity (pIC50) and predict activity (pIC50). (a) MLR, (b) SVM, and (c) BNT.

The three prediction models were established from the relations of the test set and training set through MLR, SVM, and BNT (Figure 1). The square correlation coefficients (*R* ^2^) of these three models were 0.7773, 0.851, and 0.8439, which indicates confidence in these models. The ligands which achieved the top three docking scores from the compounds, having higher bioactivity than the control, were selected and identified as candidate compounds (Table 1).

**Figure 2:**
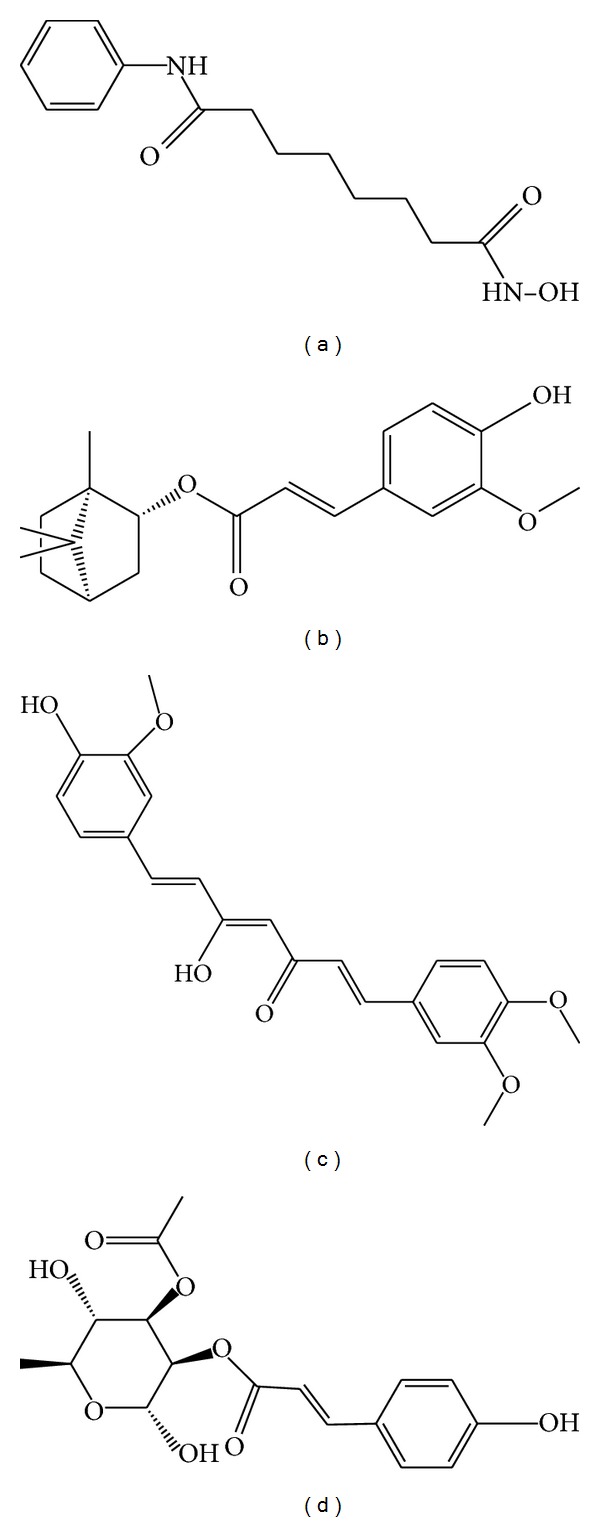
The 2D structure of control and candidate TCM compounds. (a) Control-saha, (b) (−)-Bontl ferulate, (c) monomethylcurcumin, and (d) ningposides C.

**Figure 3:**
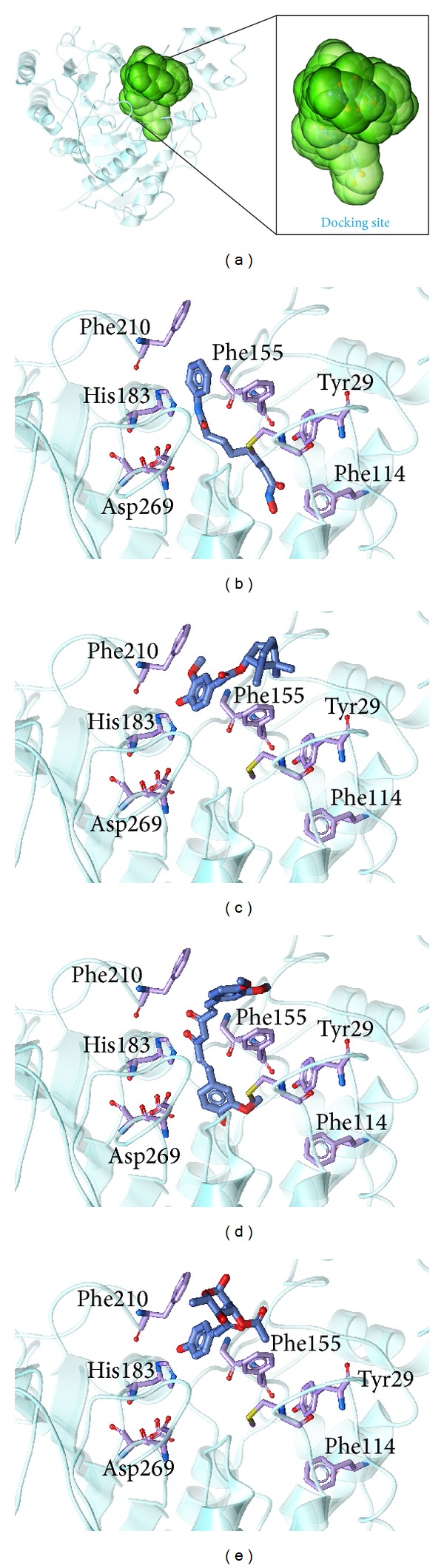
The docking poses of HDAC2. (a) HDAC2, (b) control-saha (c) (−)-Bontl ferulate, (d) monomethylcurcumin, and (e) ningposides C.

**Figure 4:**
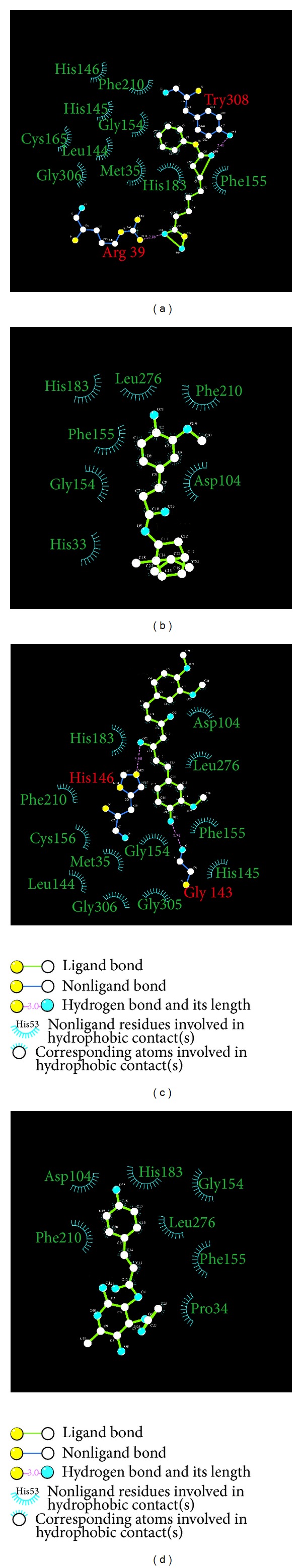
Ligplot illustrates the hydrophobic interactions. (a) Control-saha, (b) (−)-Bontl ferulate, (c) monomethylcurcumin, and (d) ningposides C.

The ligand structure (Figure 2) and the docking poses (Figure 3) of these candidate compounds were prepared for MD. From the above description, the docking poses of the candidate compounds indicated that the ligands had interactions with different important amino-acids in the protein. We determined the ligand interactions with the amino-acids by ligplot v.2.2.25 [58] (Figure 4). From this figure, we found that all the ligands can interact with Gly154, His183, and Phe210 of HDAC2. Phe155 and Leu276 are effected by selected ligands (do not have control). This indicates that the docking site could have interactions with important amino-acids and simulate the compound's target in HDAC2.

### 3.3. Molecular Dynamics Simulation

**Figure 5:**
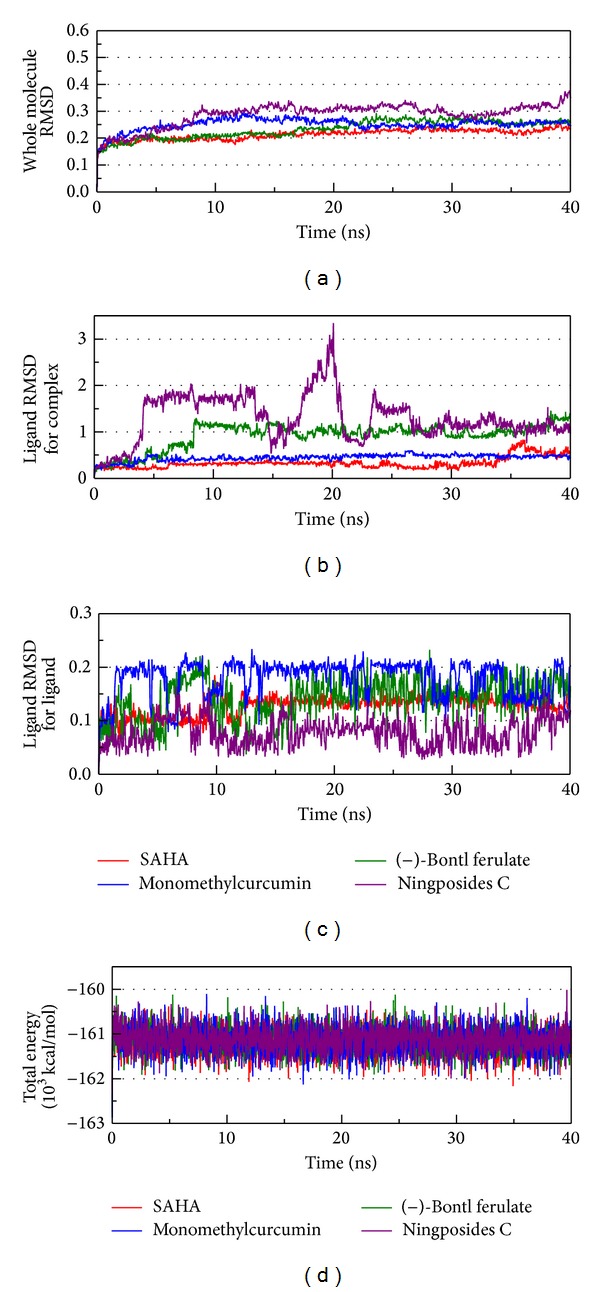
Measures of the MD trajectories. (a) Complex RMSD, (b) ligand RMSD focus on complex, (c) ligand RMSD focus on ligand, and (d) the total energy.

After 40 ns MD, the total energy and RMSD of the whole complex were recorded, with these data being drawn by OriginPro 8.5 (Figure 5). The amplitude of energy, as illustrated by the total energy of these complexes, tended to the region of −672000~−674000 kcal/mol. The RMSD of the whole complex presented by these protein and ligand interactions will tend to balance ningposides C. From Figure 5, it can be seen that ligand RMSD1 focused on ligand structure variation during the MD process, and ligand RMSD2 described the ligand variation of the whole complex. We analyzed the ligand based on the comparison between ligand RMSD1 and ligand RMSD2.

The amplitude of monomethylcurcumin in the ligand RMSD1 was the greatest, between 0.1 and 0.2, while the amplitude in the ligand RMSD2 was smooth. From this result, monomethylcurcumin had a strong interaction with HDAC2, but the complex was stable. Ningposides C had the greatest amplitude in ligand RMSD2, and this impossible variation suggested that it was not only the protein and ligand interaction, but the complex might be separated.

The site was closed to the H-bond produced site and will be twisted in torsion analysis, as seen in Figures 6(a)-(1), 6(b)-(8), 6(c)-(12), and 6(c)-(13). However, the ligand still needs interactions with the protein by H-bond production. Although the main structure of the ligand was stable, Figures 6(c)-(14) and 6(d)-(22) described the large variation of ligand structure during the MD process. This phenomenon may be caused by two hexagonal ring structures limiting the flexibility of ligand variation during the interaction.

**Figure 7:**
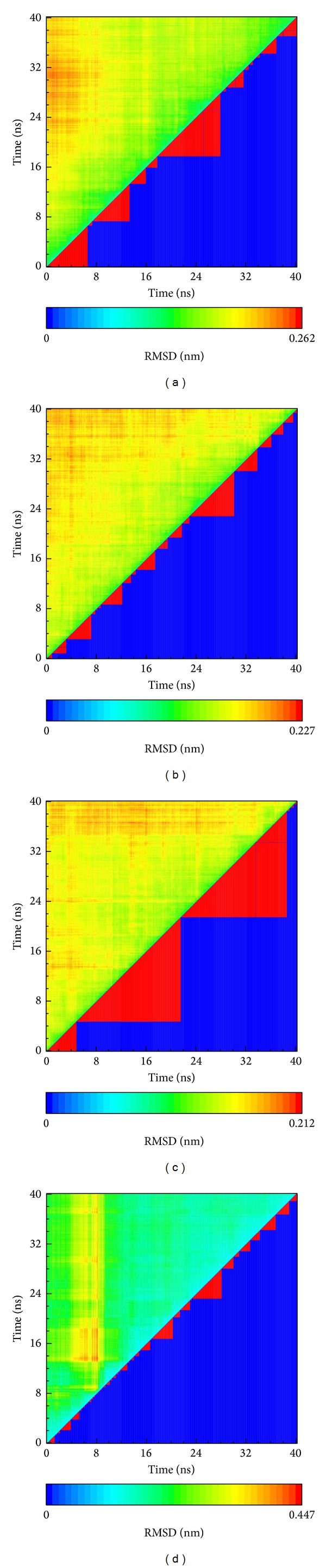
Clustering of RMSD. (a) Control-saha, (b) (−)-Bontl ferulate, (c) monomethylcurcumin, and (d) ningposides C. In each upper triangle, the color shows the RMSD difference between times on both the *x*-axis and *y*-axis. In the lower triangle, the red triangle shows the same groups based on similar RMSD.

In the clustering based on RMSD variation (Figure 7), monomethylcurcumin had the fewest groups of these compounds, indicating that the variation was lower than in the others. Ningposides C had the greatest RMSD range and the most groups of these compounds. From this result, we suggest that the complex of ningposides C and HDAC2 was unstable and the interaction of this complex seemed to be incessant. This phenomenon was described after the screening of the MD process which recorded ningposides C drifting away from the docking site at 4.12 ns, then retargeting at 14.8 ns. It drifts away from the docking site again at 16.36 ns and targets again at 21.06 ns. This result suggests that ningposides C might not be an appropriate compound due to the possibility of its moving away during MD.

**Figure 8:**
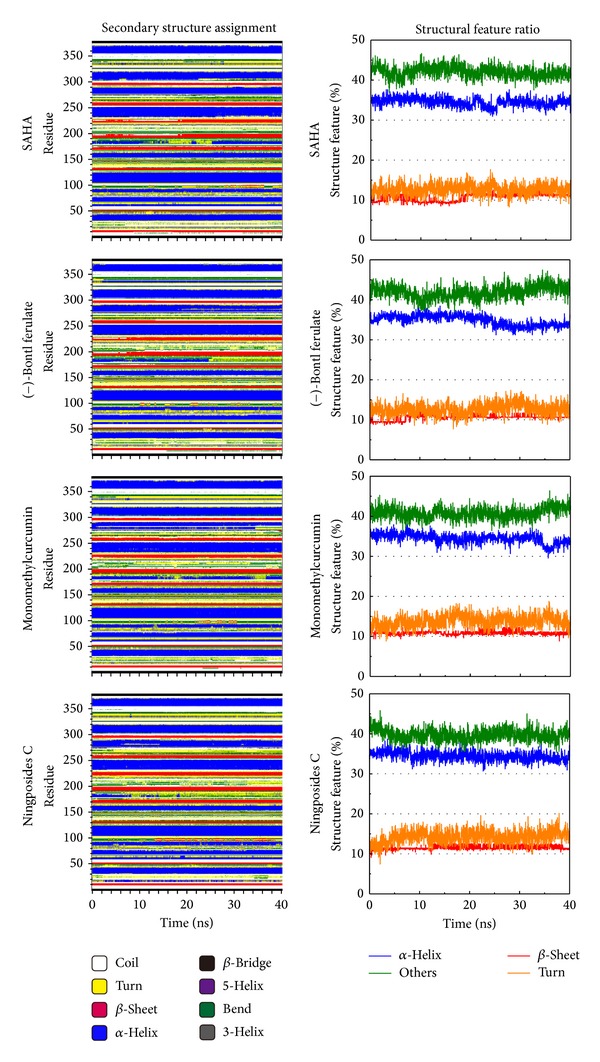
The DSSP of the protein component. DSSP shows the composition variation of the protein structure during MD.

The DSSP describes the type of protein structure (Figure 8). In this figure, SAHA causes the HDAC2 structure to reduce other types (not comprising *α* helix, *β* sheet, and turn structure) but increases the percentage of *α* helix, *β* sheet, and turn at least 20 ns interaction. (−)-Bontl ferulate, monomethylcurcumin, and ningposides C make different effect from SAHA in that the other type was increased and the *α* helix was decreased. This result suggests that these candidate compounds may result from different level effects on the amino-acids of HDAC2.

**Figure 9:**
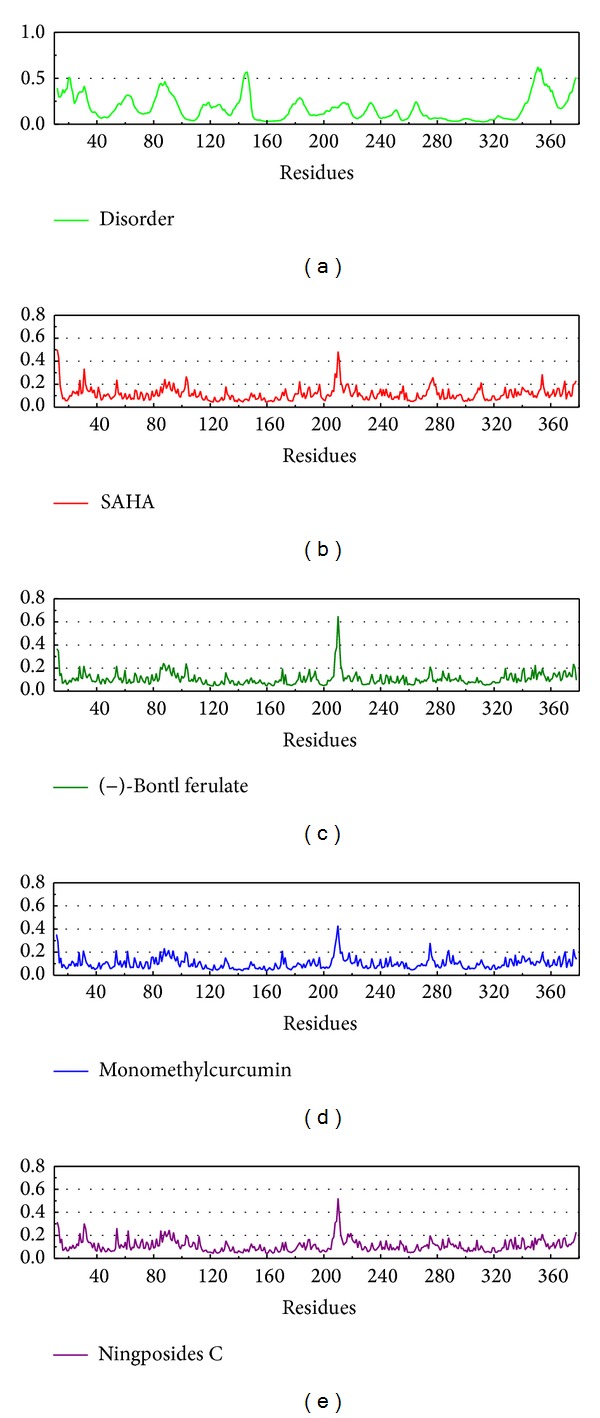
The disorder region prediction and RMSF detection. The disorder region could define the efficacy of docking based on the character of docking site and important region. For these RMSF curve is the calculation of the RMSD average for the whole MD focus on each residue.

**Figure 10:**
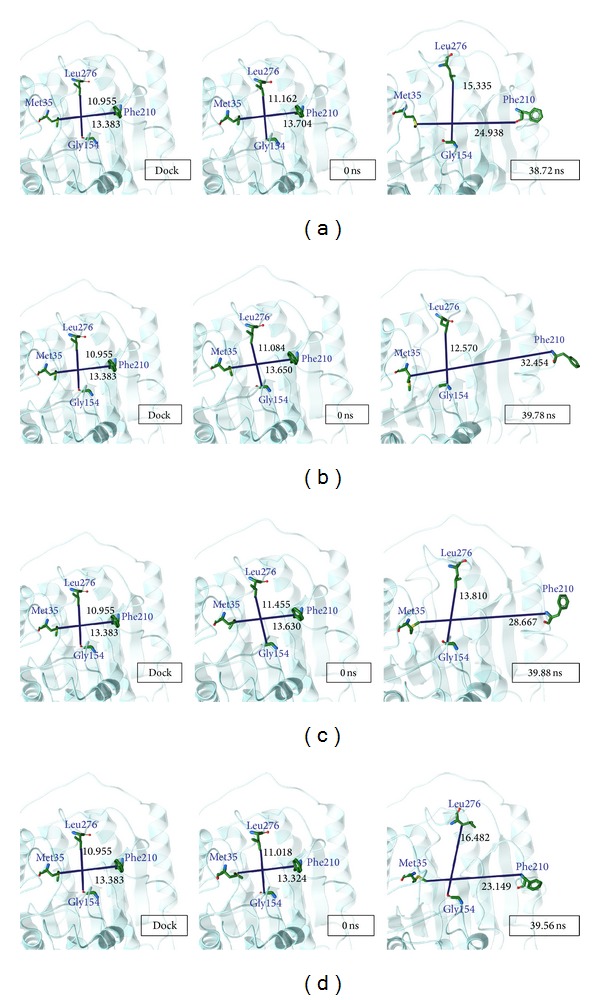
HDAC2 structure variation by ligand iteration. (a) Control-saha, (b) (−)-Bontl ferulate, (c) monomethylcurcumin, and (d) ningposides C.

The disorder shows the protein unfolding region and the RMSF describes the protein variation (Figure 9). The disorder analyzed the complete HDAC2 amino-acid sequence and this figure focuses on the region of HDAC2 structure. In this result, the residue with the larger pick is not disorder region. Thus, the disorder region has weak effect on this investigation. The RMSF recorded the amino-acid variation in 40 ns. The amplitude in RMSF among different ligand interactions was similar and this result indicates that the protein structure variation may be similar. This suggestion may be based on the structure that illustrates their matrix (Figure 10). The highest pick in RMSD was Phe210, which is one of the important amino-acids, and this result may indicate that Phe210 will move away from the ligand to inhibit the protein function.

**Figure 11:**
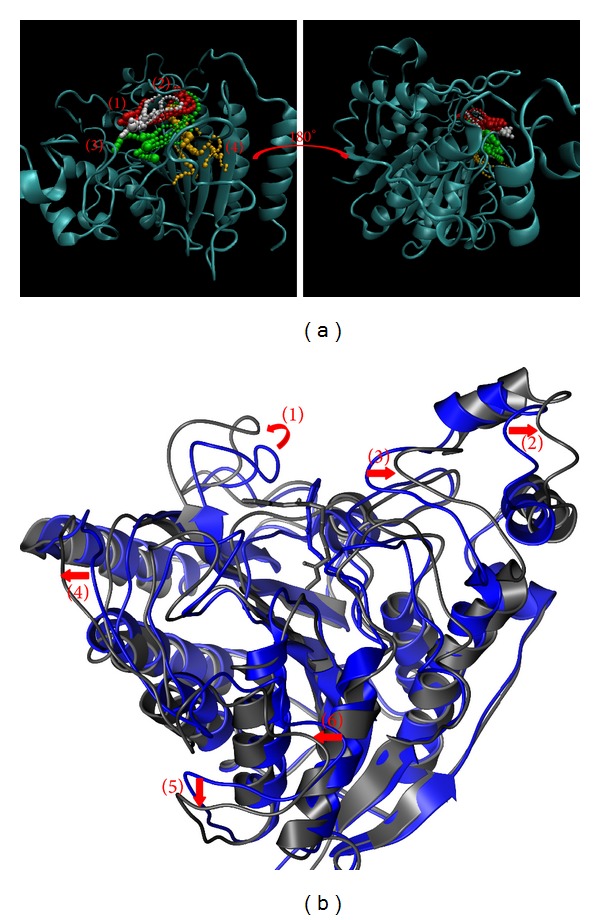
The pathway determination and HDAC2 structure variation with SAHA iteration. (a) Pathway and (b) structure variation.

The variation in structure was calculated from the centroid distance from Met35, Gly154, Phe210, and Leu276, which are four important amino-acids in HDAC2. The centroid distance between Met35 and Phe210 and the centroid distance between Gly154 and Leu276 could help us to investigate the HDAC2 variation (Figure 11). The distance between Met35 and Phe210 in SAHA increased from 13.383 to 24.938. The distance between Met35 and Phe210 changed to 32.454 in (−)-Bontl ferulate, 28.667 in monomethylcurcumin, and 23.149 in ningposides C. There was an obvious increase in this distance when the protein and ligand interacted during the MD. On the other hand, the distance between Gly154 and Leu276 in SAHA increased from 10.955 to 15.335, and this distance increased to 12.570, 13.810, and 16.482 during other ligand interactions. These distance variations the candidate ligands made during interactions were similar to SAHA, a result which can be confirmed from Figure 10.

**Figure 12:**
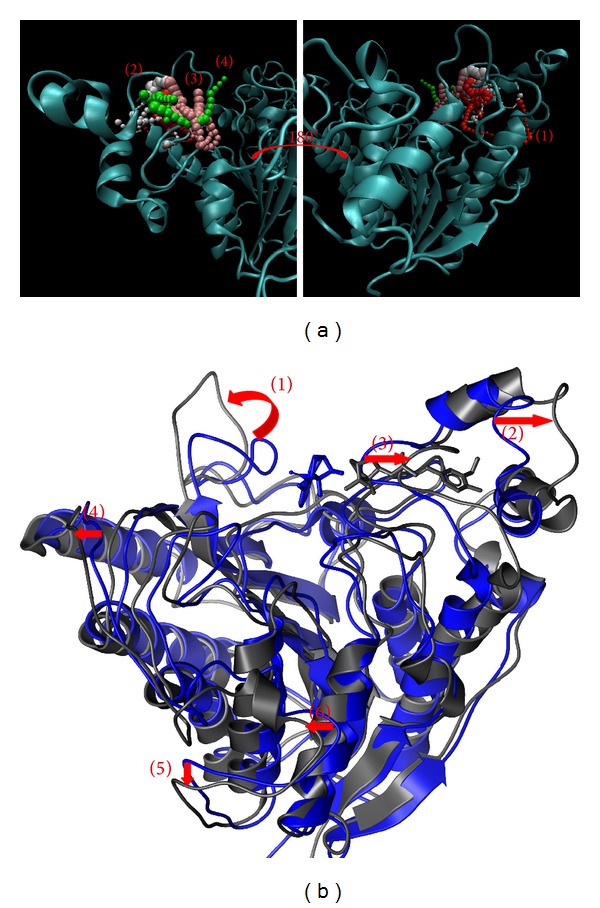
The pathway determination and HDAC2 structure variation with (−)-Bontl ferulate iteration. (a) Pathway and (b) structure variation.

**Figure 13:**
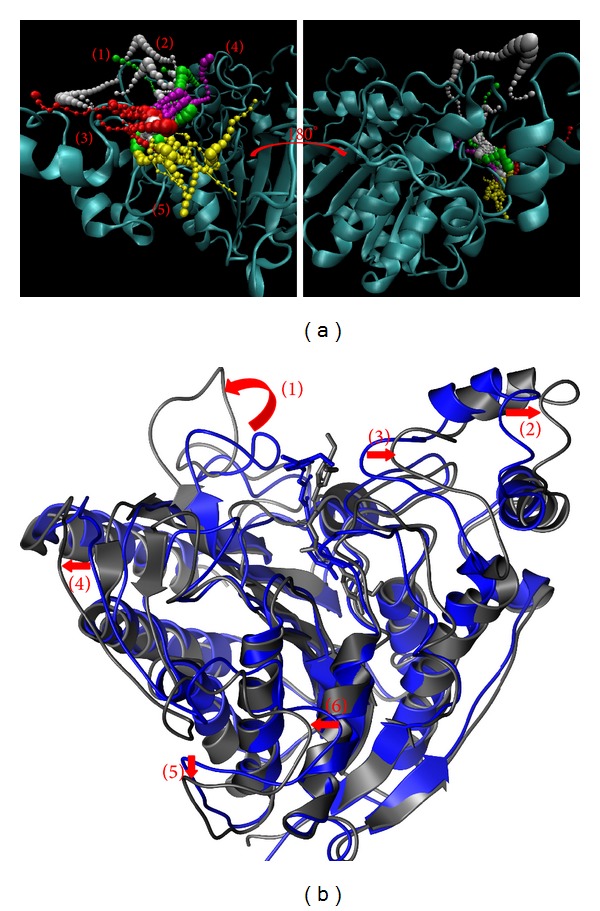
The pathway determination and HDAC2 structure variation with monomethylcurcumin iteration. (a) Pathway and (b) structure variation.

**Figure 14:**
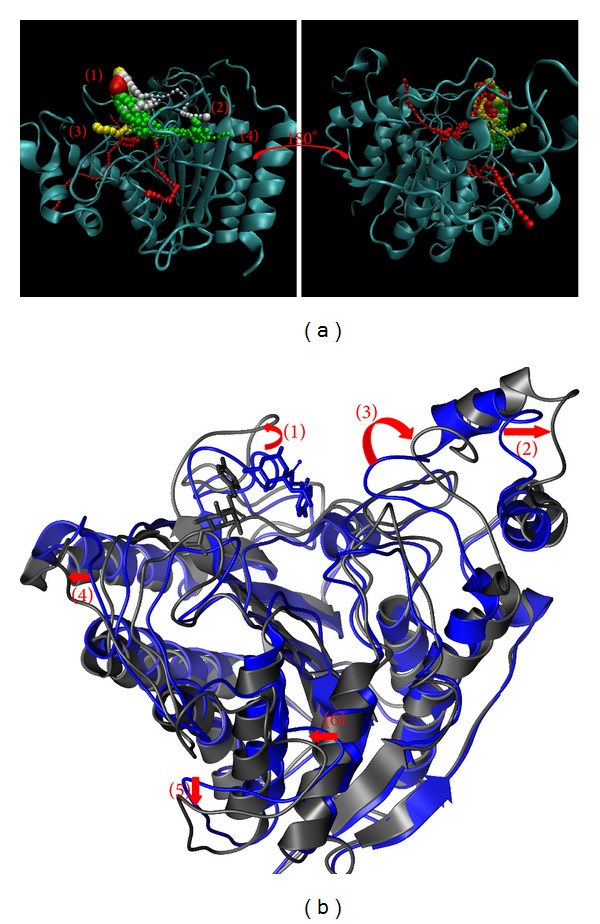
The pathway determination and HDAC2 structure variation with ningposides C iteration. (a) Pathway and (b) structure variation.

The pathway and structure variation could help to discuss the protein-ligand interaction. The pathway definition is using caver 3.0 to determine the interpath protein path during MD simulation [59] and structure variation comparing the difference between MD 0 ns and MD 40 ns (Figures 11 to 14).

While protein interacts with SAHA, the pathway for ligand was around docking site (Figure 11(a)) and the pole of HDAC2 will be spread (Figure 11(b)). Similar to SAHA, (−)-Bontl ferulate also makes the same variation in both pathway and structure (Figure 12). The pathway for monomethylcurcumin is defined around binding site based on calculation (Figure 13). One pathway for ningposides C is defined inside the protein colored in red, but that is not reasonable in biology. This situation is caused from the structure variation and then the path which is large enough for calculation. Thus, this unreasonable pathway does not take reference for the analysis of the ligand moving.

The structure variation of HDAC2 might make ligand move away from docking site if the force of target is much less. If the ningposides C, selected as candidate compounds, away from binding site is unreasonable for drug design, the MD simulation for the investigation of protein-ligand interaction is more important than docking. This situation could present that MD could confirm protein-ligand interaction by computational biology.

## 4. Conclusion

The analysis of HDAC2 found that the candidate compounds had a HDAC structure variation similar to SAHA. Because these compounds had better bioactivity than SAHA based on SVM, MLR, and BNT, these compounds had a different effect on the amino-acids and caused changes to the component percentage of HDAC2 structure. Finally, from a comparison of HDAC2 structure variation, component, and protein-ligand interactions, we propose that the candidate compounds are appropriate to inhibit HDAC2, including ningposides C due to its unstable interaction.

## Figures and Tables

**Figure 6:**
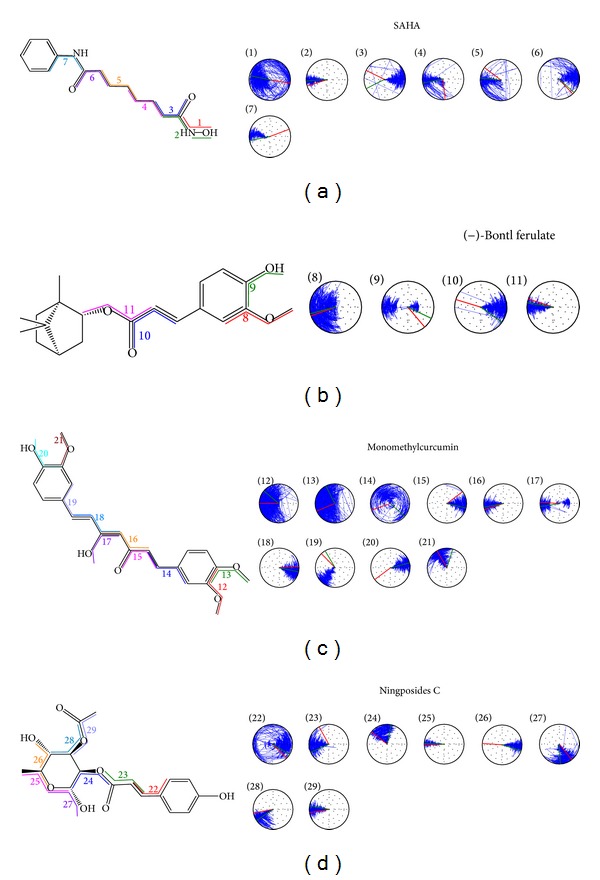
Torsion angles during MD. Torsion angle measure is designated by the number which corresponds to the radar chart. The red, green, and blue lines in the radar chart indicate the angle during docking, 0 ns, and the period of MD.

**Table 1:** 

Name	Dock score	SVM	MLR	BNT
Ningposides C	30.26	6.62067	6.88967	5.657169
Monomethylcurcumin	27.295	7.59112	6.106833	6.471242
(−)-Bontl ferulate	21.203	7.51855	7.773727	5.627211
Yakuchinone B	19.121	7.42844	5.443937	5.297163
Spinacetin	15.077	6.82774	5.493229	5.369466
SAHA∗	**3.51**	**6.61107**	**4.949132**	**4.500999**

*Control.
